# RACE/ETHNICITY DISPARITY FOR PEOPLE WITH LTSS NEEDS IN CALIFORNIA: DISABILITY, FINANCE, AND HEALTH AND WELL-BEING

**DOI:** 10.1093/geroni/igad104.0995

**Published:** 2023-12-21

**Authors:** Lei Chen

**Affiliations:** UCSF Philip R. Lee Institute for Health Policy Study, San Francisco, California, United States

## Abstract

Older adults and people with disabilities have high needs for Long-Term Services and Supports (LTSS). Disability status, financial difficulties, and health and well-being are likely not uniformly experienced by all people with LTSS needs. Subgroups of older adults and people with disabilities, such as racial/ethnic minorities, may be at significant risk of experiencing financial difficulties and stress about making ends meet. This study used the first cycle of data (2019-2020) from the California Long-Term Services and Supports (CA-LTSS) survey, merged with select data from the California Health Interview Survey (CHIS) (N = 2,030). Multivariate Regressions and Conditional Process Analysis (CPA) were applied to test the hypothesized relationships. Findings show that black/African American and Asian participants with LTSS needs were more likely to have cognitive impairments. Black/African American participants with LTSS needs were more likely to report difficulties in activities of daily living. Racial/ethnic minorities with LTSS needs experienced diverse and more financial difficulties on average than their white counterparts. Asian participants with LTSS needs reported worse psychological distress than other racial/ethnic groups. American Indians and Alaska Natives with cognitive impairments were more likely to have more financial difficulties, and those with difficulty in instrumental activities of daily living were more likely to report worse self-rated health. This study moves beyond the typical white/black disparities analyses and focuses on diverse racial/ethnic groups. We need to address the financial needs of various racial/ethnic groups of people with LTSS needs and target policies and service programs to meet their mental health needs.

